# Vitamin C promotes apoptosis in breast cancer cells by increasing TRAIL expression

**DOI:** 10.1038/s41598-018-23714-7

**Published:** 2018-03-28

**Authors:** David W. Sant, Sushmita Mustafi, Christopher B. Gustafson, Joshua Chen, Joyce M. Slingerland, Gaofeng Wang

**Affiliations:** 1 John P. Hussman Institute for Human Genomics, Dr. John T. Macdonald Foundation Department of Human Genetics, University of Miami Miller School of Medicine, Miami, FL 33136 USA; 2 Braman Family Breast Cancer Institute, University of Miami Miller School of Medicine, Miami, FL 33136 USA; 3 Sylvester Comprehensive Cancer Center, University of Miami Miller School of Medicine, Miami, FL 33136 USA

## Abstract

Genomic loss of 5-hydroxymethylcytosine (5hmC) accompanies malignant cellular transformation in breast cancer. Vitamin C serves as a cofactor for TET methylcytosine dioxygenases to increase 5hmC generation. Here we show that the transcription of SVCT2, a major vitamin C transporter, was decreased in human breast cancers (113 cases) compared to normal breast tissues from the same patients. A decreased SVCT2 expression was also observed in breast cancer cell lines. Treatment with vitamin C (100 μM) increased the 5hmC content in MDA-MB-231 breast cancer cells and markedly altered the transcriptome. The vitamin C treatment induced apoptosis in MDA-MB-231 cells, which was verified in two additional breast cancer cell lines. This pro-apoptotic effect of vitamin C appeared to be mediated by TRAIL, a known apoptosis inducer. Vitamin C upregulated TRAIL transcripts (2.3-fold increase) and increased TRAIL protein levels. The upregulation of TRAIL by vitamin C was largely abolished by siRNAs targeting TETs and anti-TRAIL antibody abrogated the induction of apoptosis. Furthermore, the apoptosis promoted by vitamin C was associated with Bax and caspases activation, Bcl-xL sequestration, and cytochrome c release. Taken together, these results suggest a potential role of physiological doses of vitamin C in breast cancer prevention and treatment.

## Introduction

Aberrant epigenetic alterations, which reflect the interface of a dynamic microenvironment and the genome are involved in malignant cellular transformation^1^. Global loss of 5-hydroxymethylcytosine (5hmC) has been recognized as an epigenetic hallmark in most, if not all, types of cancer including breast cancer^2^. 5hmC content is relatively high in normal breast epithelial cells, but shows a progressive loss in breast cancers^3–6^. 5hmC is converted from 5-methylcytosine (5mC) as an initial step of active DNA demethylation, which is catalyzed by ten-eleven translocation (TET) methylcytosine dioxygenases^7^. TETs can further oxidize 5hmC to 5-formylcytosine (5fC) and 5-carboxylcytosine (5caC), which are eventually replaced by unmodified cytosine, thus completing the process of active DNA demethylation^8^. 5hmC, which is relatively stable, recruits different sets of binding proteins and exerts distinct effects on transcription compared to 5mC^8^. Thus, in addition to being a DNA demethylation intermediate, 5hmC also serves as an epigenetic mark with unique regulatory functions. The global loss of 5hmC could change DNA methylation-demethylation dynamics and gene transcription, further leading to a cascade that drives phenotypic transformation from normal breast epithelial cells to breast cancer cells.

Loss of 5hmC within primary breast cancers is a biomarker of poor prognosis^9^, raising the possibility that increasing 5hmC might offer a novel therapy for breast cancer. In a small subset of breast cancers, loss of 5hmC occurs via decreased TET1 expression^10^. It has been shown that overexpression of TET1 can partially re-establish a normal 5hmC profile in breast cancer cells and decrease their invasiveness^10^. While overexpressing TET1 using viral vectors in patients might not be clinically feasible, this discovery suggests that restoring normal 5hmC content may have therapeutic potential for breast cancer.

TETs belong to the iron and 2-oxoglutarate (2OG)-dependent dioxygenase superfamily, which catalyzes the hydroxylation of a diverse variety of substrates. These dioxygenases utilize Fe(II) as a cofactor, 2OG as a co-substrate, and some of them require vitamin C as an additional cofactor for full catalytic activity. Vitamin C (L-ascorbic acid) exists predominantly as the ascorbate anion under conditions of physiological pH. We and others recently showed that vitamin C, which has the capacity of reducing catalytic inactive Fe(III) to catalytic active Fe(II), upregulates the generation of 5hmC by acting as a cofactor for TET to hydroxylate 5mC^11–15^. This novel function of vitamin C to modulate DNA demethylation prompted us to test whether vitamin C treatment might upregulate TET action and have effects similar to TET overexpression in breast cancer cells. Here, we show that decreased expression of sodium-dependent vitamin C transporter 2 (SVCT2), appears to mediate the loss of 5hmC in breast cancer, despite stable TET expression. Treatment with vitamin C increases 5hmC content in breast cancer cells, changes the transcriptome, and induces apoptosis by increasing expression of the apoptosis inducer gene, TNF-related apoptosis-inducing ligand (TRAIL).

## Results

### Vitamin C transporter is downregulated in primary human breast cancer

**Figure 1:**
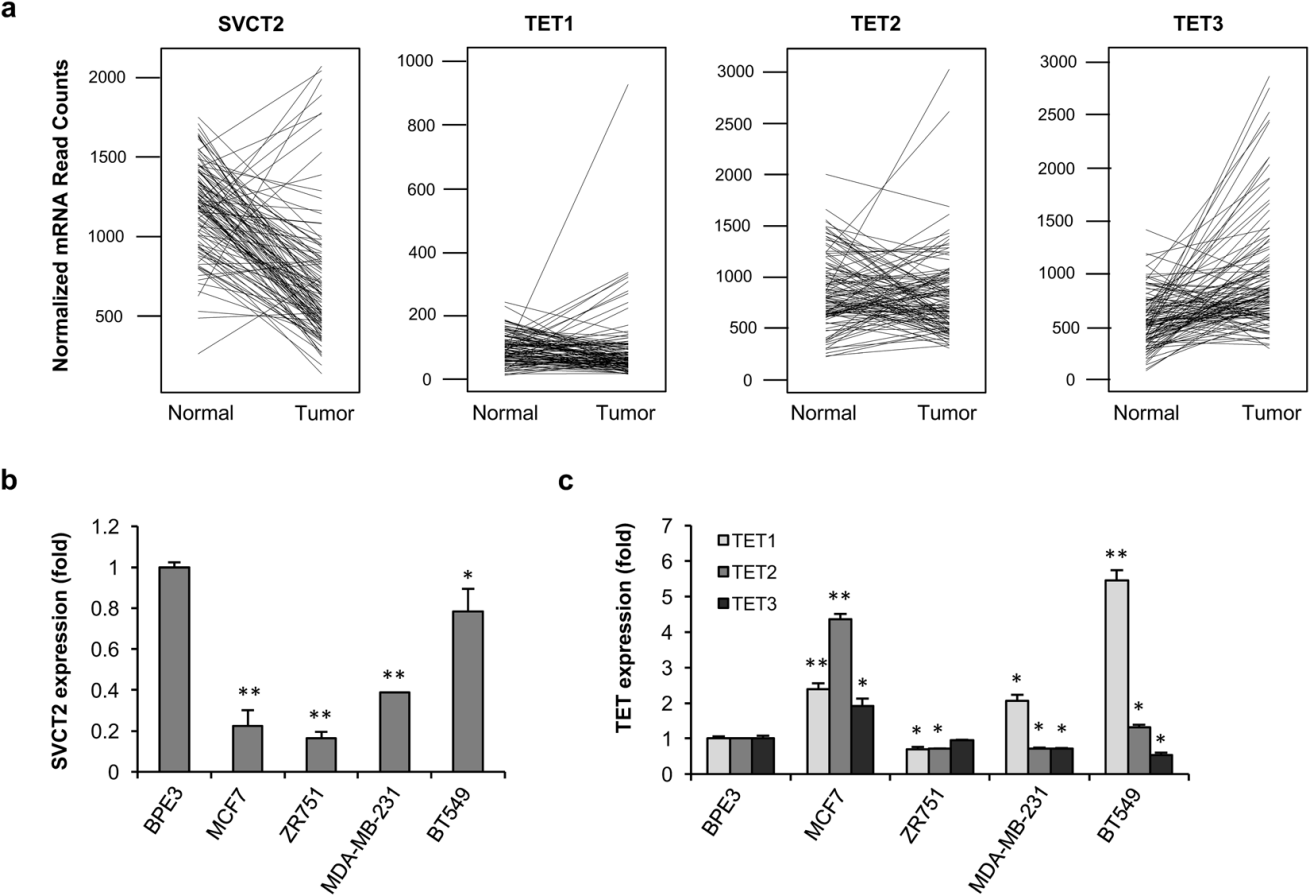
Downregulation of SVCT2 in breast cancer. (**a**) RNA-seq of 113 paired normal breast tissue/breast cancer available in the TCGA dataset shows that the mRNA levels of SVCT2 are decreased in many cancer samples compared to controls (P = 2.31 × 10^−19^). In contrast TETs remain unchanged or increased in the majority of breast cancer cases. Each line represents an individual patient, linking the level of mRNA in the normal tissue and tumor tissue from that specific patient. (**b**) Quantitative RT-PCR shows that SVCT2 mRNA levels were decreased in breast cancer cell lines. (**c**) The mRNA level of TETs was either increased or decreased in breast cancer cell lines shown by quantitative RT-PCR.

Our recent work has indicated that vitamin C promotes 5hmC generation by serving as a cofactor for TETs^11,12^. Intracellular vitamin C deficiency would fail to maintain the catalytic activity of TETs, resulting in the loss of 5hmC as observed in breast cancer^3–6^. To identify potential factors responsible for the observed loss of 5hmC in primary human breast cancers, we analyzed RNA-seq data from The Cancer Genome Atlas (TCGA). This dataset contained 113 matched pairs of breast cancer and normal breast tissue obtained from the same patients. Vitamin C enters and accumulates in breast epithelial cells mainly via SVCT2, which is encoded by the solute carrier family 23 member 2 gene (*SLC23A2*)^16^. The expression of SVCT2 in breast cancer was decreased compared to normal breast epithelium (*P* = 2.31 × 10^−19^, Fig. 1a). Of the 113 breast cancer samples, the SVCT2 expression was decreased in 72.5% (n = 82) by at least 1.5 fold compared to the matched normal breast tissues. TET1, which was expressed at a very low level, was slightly downregulated (0.67 fold) in 42.5% of breast cancer cases. TET2 and TET3 were downregulated in only 28% and 8.9% of cases, respectively. In most breast cancers, TET1, TET2, and TET3 expression levels were unchanged or even increased compared to their matched normal tissues, as shown in Fig. 1a. These data suggest that the reduced SVCT2 expression, rather than that of TETs, might be a major cause for the loss of 5hmC observed in a majority of breast cancers.

SVCT2 expression was then evaluated in the hTERT-immortalized, normal human breast epithelial line (BPE3) and in four breast cancer lines: estrogen receptor positive (ER^+^)/progesterone receptor positive (PR^+^) luminal MCF7, ER^+^/PR^−^ luminal ZR751, and triple negative (ER^−^/PR^−^/HER2^−^) basal lines, MDA-MB-231 and BT549. SVCT2 expression was consistently decreased in each of the four breast cancer cell lines compared to BPE3 (Fig. 1b). Notably, TET expression in these breast cancer cell lines varied greatly. TET 1, 2, and 3 levels in MCF7 cells were actually increased compared to the immortalized normal breast epithelial cells (Fig. 1c). This decreased expression of SVCT2 would reduce vitamin C uptake in breast cancer cells, creating an intracellular vitamin C deficiency which could give rise to decreased 5hmC levels.

### Vitamin C treatment increases 5hmC content in breast cancer cells

**Figure 2:**
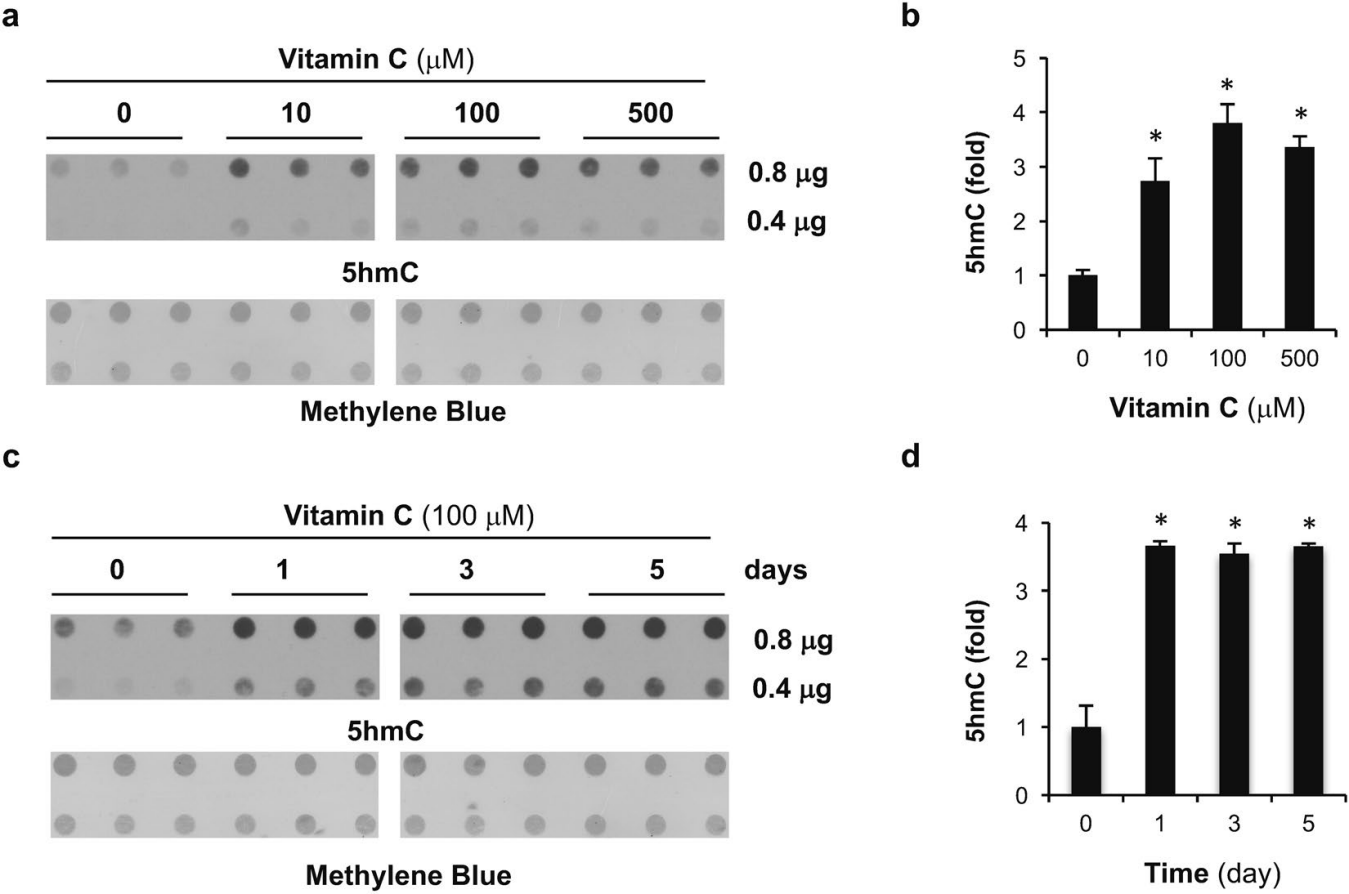
Vitamin C treatment elevates 5hmC content in breast cancer cells. (**a**) The dot-blot shows that treatment with 10, 100, and 500 μM vitamin C for 24 hours increases the global content of 5hmC. (**b**) The semi-quantitation of the dot-blot shows that 10 μM vitamin C treatment for 24 hours increases the global content of 5hmC, supplementation to 100 μM further increases 5hmC levels, but further supplementation to 500 μM does not increase the levels further. (**c**) The dot-blot shows that treatment with 100 μM vitamin C increases the global content of 5hmC. (**d**) The semi-quantitation of the dot blot shows that 1 day of treatment with 100 μM vitamin C treatment increases the global content of 5hmC, and that supplementation for longer periods largely maintains the 5hmC level (* indicates *P* < 0.05).

In an effort to test whether vitamin C supplementation might restore 5hmC content in breast cancer cells by enhancing TET activity, we tested the effect of adding vitamin C to the cell culture media. We examined the effect of vitamin C on 5hmC generation in MDA-MB-231 cells, where the expression of SVCT2 is lower but the expression of TET1-3 is similar or higher than in BPE3 cells (Fig. 1b,c). Using a dot-blot assay to measure global 5hmC content, the 5hmC signal was barely detectable in MDA-MB-231 cells cultured without vitamin C (Fig. 2a). The average concentration of vitamin C in healthy human plasma is generally at ~50 μM range and can reach up to ~150 μM^17^. Supplementation of vitamin C (10 μM) in the medium for 24 hours increased the 5hmC content about 2.5 fold. A higher physiological plasma concentration of vitamin C (100 μM) further increased 5hmC by nearly 4-fold over the basal levels (Fig. 2b). Treatment with a pharmacological vitamin C concentration (500 μM) increased 5hmC generation to a similar extent as that observed with 100 μM vitamin C. Thus, vitamin C treatment at pharmacological levels, which requires intravenous injection, would not incur greater benefit in promoting 5hmC generation in breast cancer cells than vitamin C at 100 μM, which is achievable *in vivo* with oral delivery.

We then investigated the vitamin C-induced increase in 5hmC over time using 100 μM vitamin C. Treatment of MDA-MB-231 cells with vitamin C (100 μM) for 1, 3, or 5 days all caused an increase in 5hmC to nearly 4-fold of the baseline level (Fig. 2c,d). The doubling time for MDA-MB-231 cells is about 24 hours and 5hmC is not maintained during DNA replication. Thus, vitamin C at 100 μM appears to sustain an elevated 5hmC level in these rapidly growing MDA-MB-231 cells. In MDA-MB-231, TET enzymes may be poised to generate 5hmC. With an increased availability of their co-factor vitamin C, they are able to more efficiently catalyze the hydroxylation reaction. The upregulation of 5hmC content by vitamin C treatment is comparable to the effect of overexpressing TET1 in this cell line^10^.

### Vitamin C changes the transcriptome of breast cancer cells

**Figure 3:**
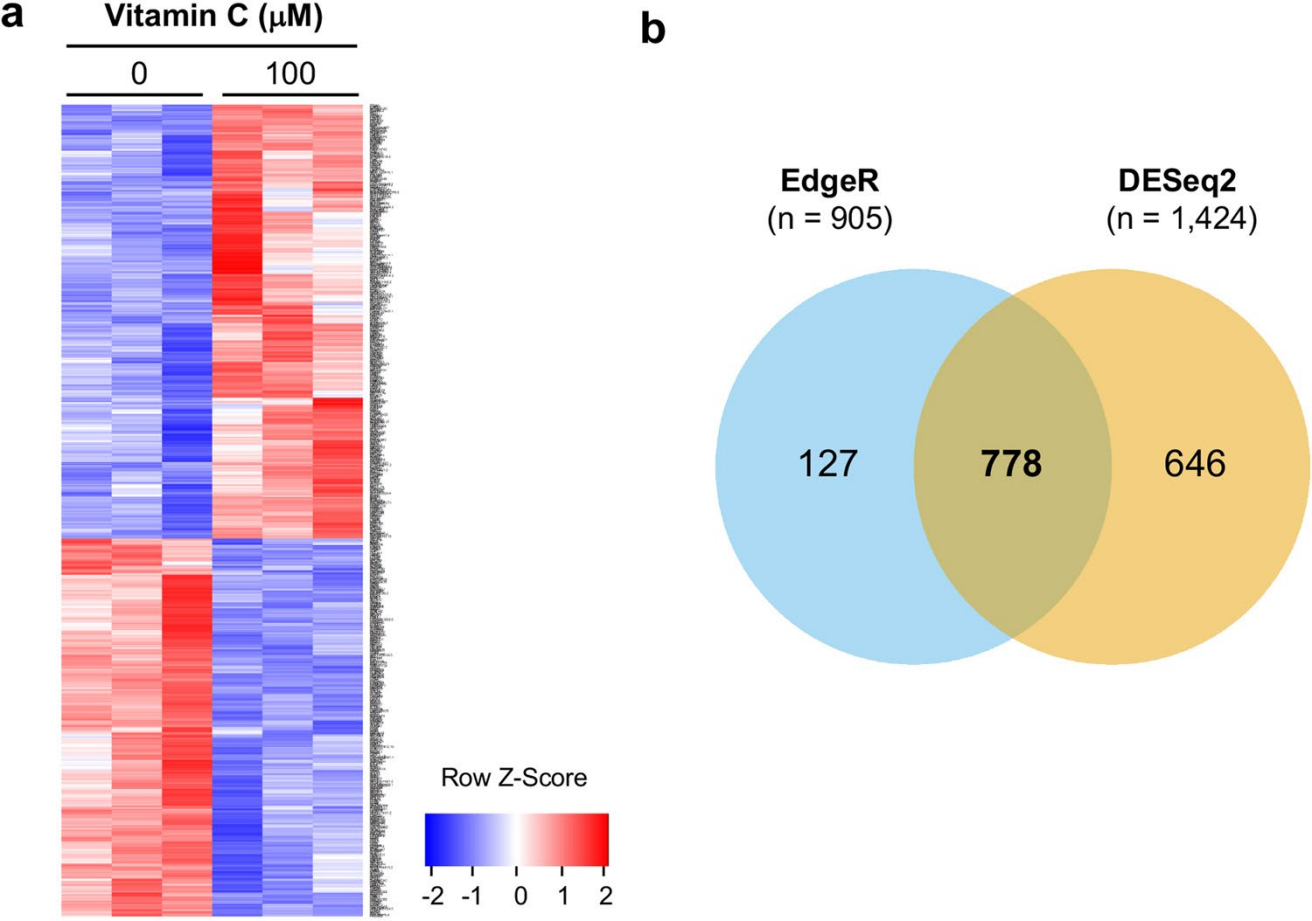
Vitamin C shifts the transcriptome of breast cancer cells. (**a**) The heatmap showing the relative transcript levels of the differential genes in MDA-MB-231 cells treated with 100 μM vitamin C or no vitamin C shows that the expression pattern changes after treatment. (**b**) Differentially expressed genes are identified by edgeR (905 genes) and DESeq. 2 (1,424 genes). 778 genes were identified by the two methods.

An increase in the global 5hmC content shifts DNA methylation-demethylation dynamics, which could consequently change gene expression profiles. Thus, we hypothesized that the vitamin C-induced global increase of 5hmC might also lead to changes in the transcriptome. Whole transcriptome sequencing, also known as RNA-seq, was undertaken to evaluate the influence of vitamin C treatment on the MDA-MB-231 transcriptome. Paired-end RNA-seq data enabled us to digitally quantifying transcript levels, and to display alternative splice variants and non-coding RNA species. MDA-MB-231 cells were treated with vitamin C at 100 μM for 3 days. DNA and RNA were simultaneously extracted from MDA-MB-231 cells cultured in the same wells (n = 3 per group). Vitamin C-induced 5hmC was confirmed by dot-blot assay (data not shown) in samples submitted for sequencing. We observed a shift in the MDA-MB-231 transcriptome after vitamin C treatment as shown by heatmap (Fig. 3a). 905 genes were determined to be differentially expressed by edgeR and 1,424 genes were determined to be differentially expressed by DESeq. 2. Of these, 778 genes were significantly and differentially expressed using both methods (Fig. 3b). Of the 778 genes, 363 genes were downregulated while 415 genes were up-regulated. The top 10 upregulated genes (*TNFSF10*, *CYP1B1*) and downregulated genes (*TFRC*, *PGK1*, *BNIP3*, *NDRG1*, *BNIP3L*, *ADM*, *PDK1*, *HK2*) were chosen for validation based on fold changes. The expression changes of all 10 genes were verified by qRT-PCR (Supplementary Table 1).

### Vitamin C upregulates the expression of TRAIL

**Figure 4:**
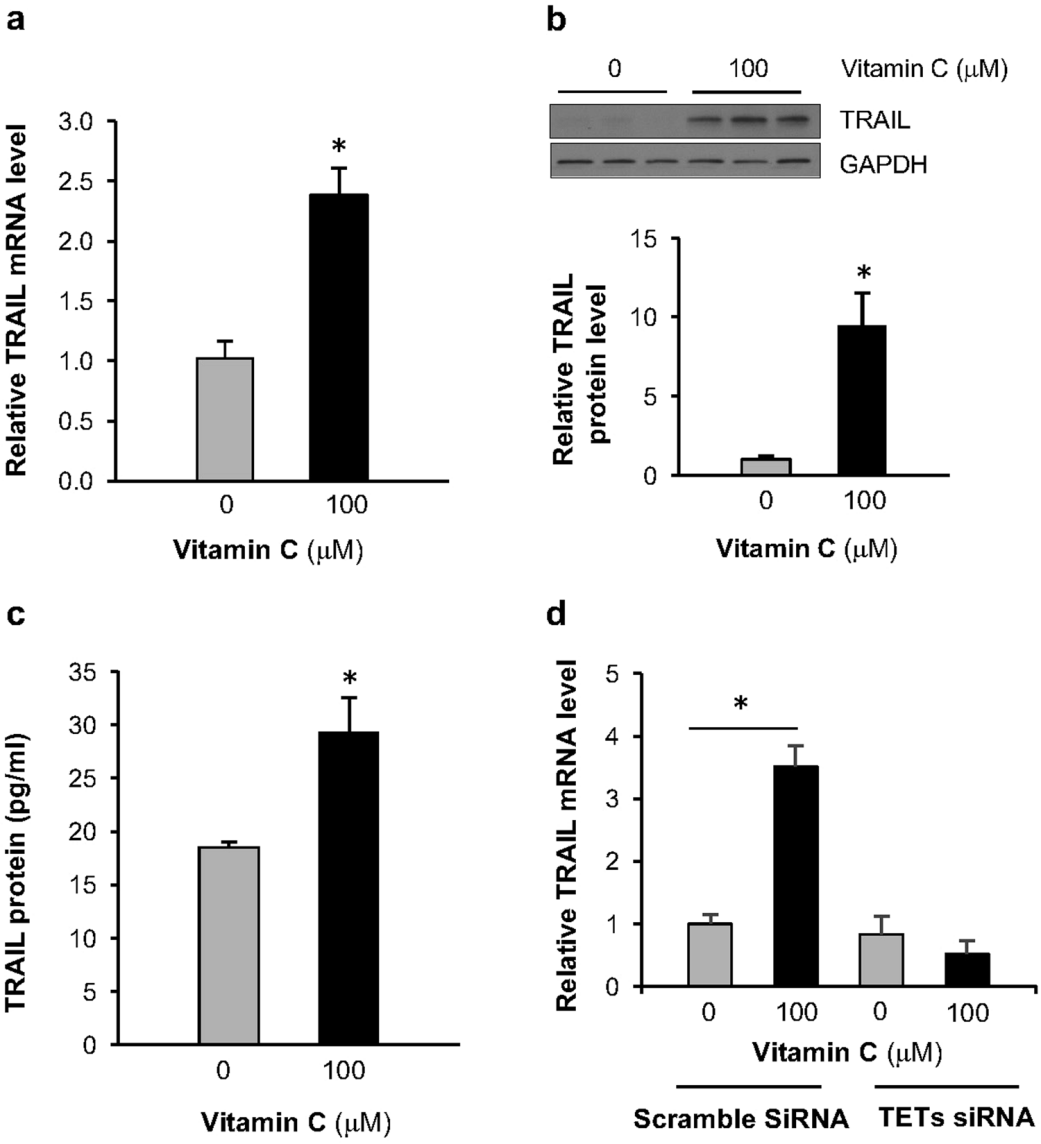
Vitamin C increases the expression of TRAIL. (**a**) The mRNA levels of TRAIL, measured by qRT-PCR, are increased in MDA-MB-231 cells treated for 3 days with 100 µM vitamin C. (**b**) The levels of TRAIL protein are increased, measured by Western blot, in MDA-MB-231 cells treated for 7 days with 100 µM vitamin C. (**c**) The secretion of TRAIL protein is increased, measured by ELISA test, in the culture media of MDA-MB-231 cells treated for 3 days with 100 µM vitamin C. (* indicates P < 0.05). (**d**) MDA-MD-231 cells transfected with scramble RNAi show an increase in TRAIL mRNA when treated with Vitamin C, but when transfected with TETs RNAi, vitamin C treatment does not elevate the TRAIL mRNA levels.

One of the most dramatic changes in transcription was *TNFSF10*, which exhibited a ~2.3-fold increase after 3 days exposure to vitamin C. *TNFSF10* encodes the TNF-related apoptosis-inducing ligand (TRAIL), a potent inducer of apoptosis that is a therapeutic target in many types of cancer including breast cancer^18^. The increased mRNA, protein, and secretion of *TNFSF10* following vitamin C (100 μM) treatment was confirmed in MDA-MB-231 cells (Fig. 4a–c, Supplementary Figure 1). The change in *TNFSF10* expression was consistent with both sodium ascorbate and L-ascorbic acid, but was not observed when the cells were treated with glutathione, a potent antioxidant with no effect on DNA demethylation (Supplementary Figure 2). To further evaluate whether the upregulation of TRAIL results from a vitamin C-stimulated increase in TET-dependent DNA demethylation, we utilized siRNA to knockdown the expression of all 3 TETs (TET1, TET2, and TET3) in MDA-MB-231 cells. In TET-knockdown cells, the TRAIL level was essentially unchanged (*P* > 0.05) by treatment with vitamin C (100 µM). In contrast, the TRAIL mRNA level was significantly increased by the same vitamin C treatment in MDA-MB-231 cells transfected with scramble siRNA (Fig. 4d). Thus, the upregulation of TRAIL expression by vitamin C appears to require the TET-mediated DNA demethylation pathway.

### Vitamin C induces apoptosis by upregulating TRAIL

**Figure 5:**
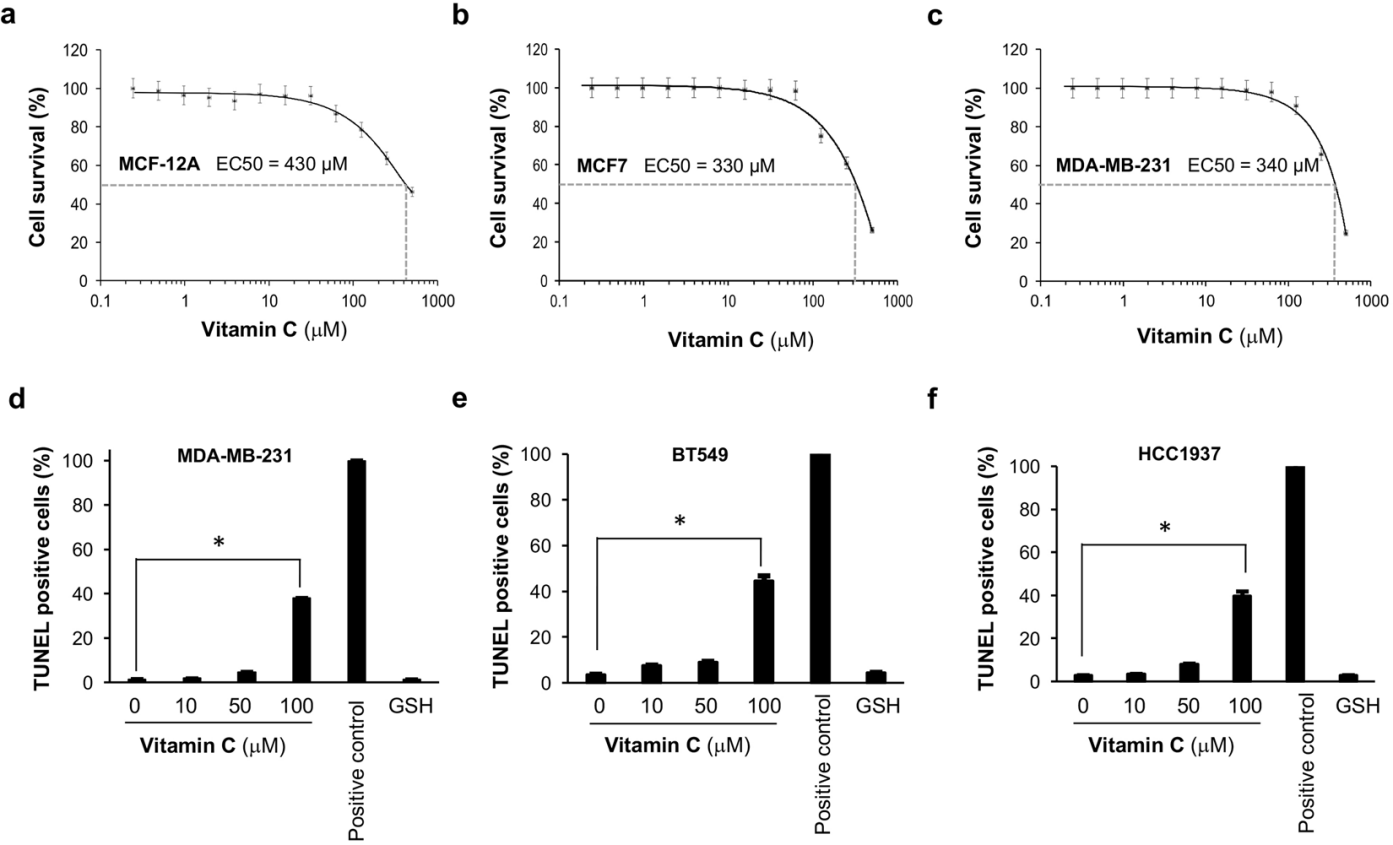
Induction of apoptosis in breast cancer cells by vitamin C. (**a**–**c**) The effect of vitamin C on cell survival shows the EC_50_ is at similar levels for healthy breast epithelium and breast cancer cells. (**d**–**f**) Apoptotic cells were measured in MDA-MB-231, BT549, and HCC1937 cell lines by colorimetric TUNEL assay after treatment with different doses of vitamin C. Vitamin C at 100 μM, but not 10 μM significantly increases apoptosis. In contrast, GSH (100 μM) has no effect on apoptosis.

To avoid the effect of protons released from ascorbic acid, we used sodium ascorbate to test breast cancer cell sensitivity to vitamin C. The EC_50_ value for vitamin C (ascorbate anion) in killing non-malignant breast epithelial cells (EC_50_ = 430 μM for non-malignant MCF-12A cells; EC_50_ = 410 μM for BPE3) was only slightly higher than breast cancer cells (EC_50_ = 330 μM for MCF7 cells; EC_50_ = 340 μM for MDA-MB-231 cells) (Fig. 5a–c). These results suggest that it might not be practical to apply high concentrations of vitamin C by intravenous injections in patients because of toxicity to healthy cells.

**Figure 6:**
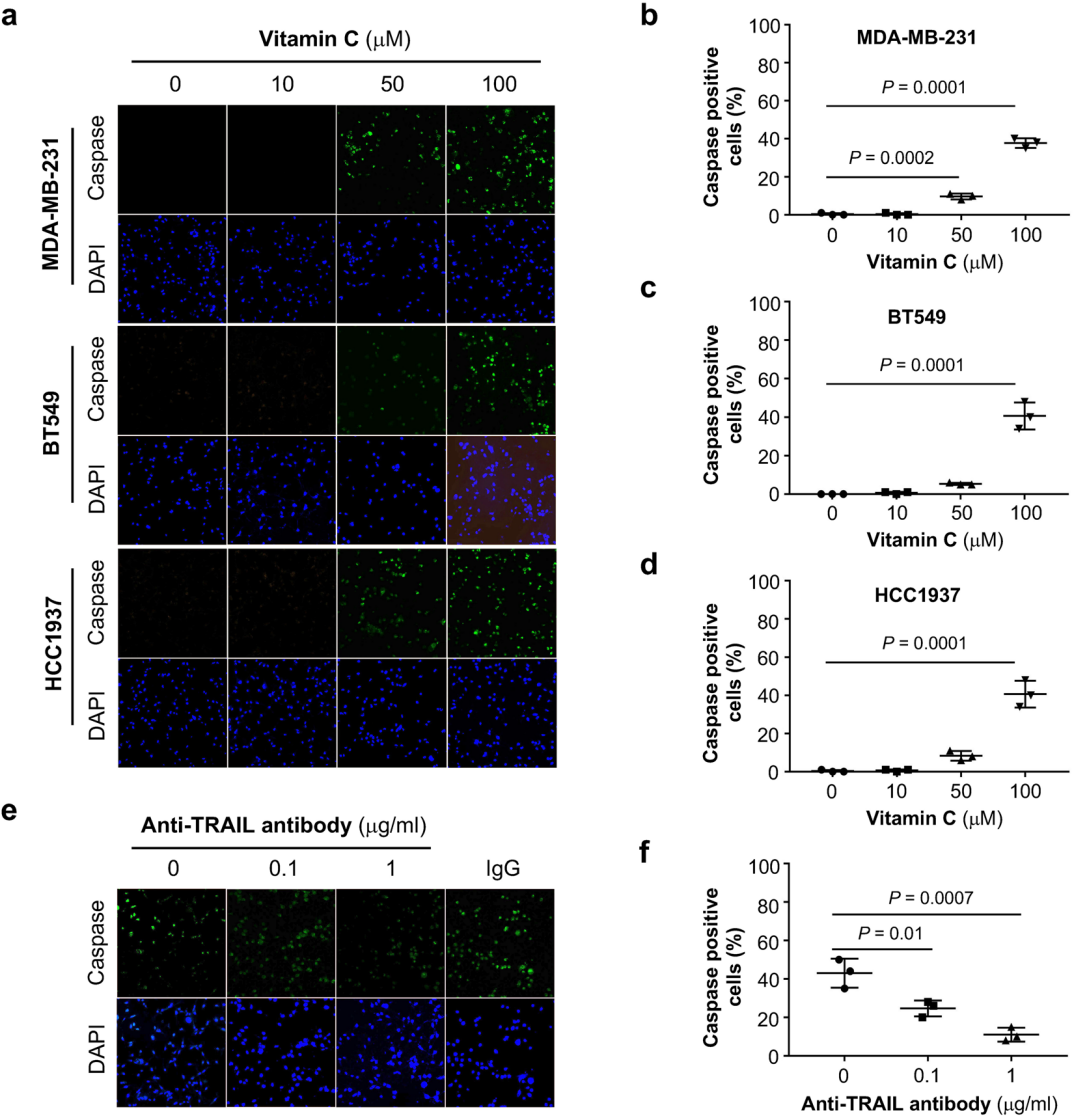
Effect of vitamin C treatment on caspase activation in breast cancer cells. (**a**) Vitamin C treatment at 100 μM significantly enhances poly caspase activation in MDA-MB-231, BT549 and HCC937 cells. (**b–d**) Quantification of caspase active cell in MDA-MB-231, BT 549, and HCC1937 cells. (**e**) Anti-TRAIL antibody blocks caspase activation in MDA-MB-231 cells treated by vitamin C (100 μM). (**f**) Quantification showing a significant decrease in caspase active cells when anti-TRAIL antibody is applied to vitamin C treated cells.

We next assayed whether levels of vitamin C that are physiologically achievable might exert an antitumor effect. As noted above, vitamin C at 100 μM increases global 5hmC as efficiently as a pharmacological concentration (500 μM) in breast cancer cells *in vitro*. We reasoned that by upregulating TRAIL, vitamin C might activate apoptosis. Treatment with vitamin C at 100 μM for 3 days, but not at lower concentrations, induced apoptosis in MDA-MB-231 cells measured by colorimetric TUNEL assay (Fig. 5d). The induction of apoptosis was then verified in two other breast cancer cell lines BT549 and HCC1937 (Fig. 5e,f). In contrast, glutathione (GSH), a general antioxidant, had no effect on 5hmC generation and did not affect apoptosis of breast cancer cells. To further confirm the induction of apoptosis by vitamin C, breast cancer cell lines including MDA-MB-231, BT549, and HCC1937 were probed for active caspase using poly caspase immunofluorescence staining on live cells. Caspase stained cells increased after 3 days treatment with vitamin C at 100 μM, but treatment at lower concentrations increased apoptosis only minimally (Fig. 6a–d). These results suggest that treatment of breast cells with vitamin C at a physiological concentration induces apoptosis and involves caspase activation. To test if apoptosis induction is TRAIL dependent, MDA-MB-231 cells were treated with an inhibitory monoclonal antibody against TRAIL in MDA-MB-231 cells during vitamin C treatment (100 μM) (Fig. 6e,f). The anti-TRAIL antibody at concentrations of 0.1 and 1 μg/mL significantly reduced apoptosis (*P* = 0.01 and 0.007) in vitamin C treated MDA-MB-231 cells where control mouse IgG did not (Fig. 6e). Furthermore, addition of TRAIL protein (0.1 μg/mL) to the media induced apoptosis in ~75% of MDA-MB-231 cells, but this effect was also largely abolished by addition of anti-TRAIL antibody to the media (*P* = 0.0001, Supplementary Figure 3). These results suggest the induction of apoptosis by vitamin C is primarily mediated by TRAIL.

**Figure 7:**
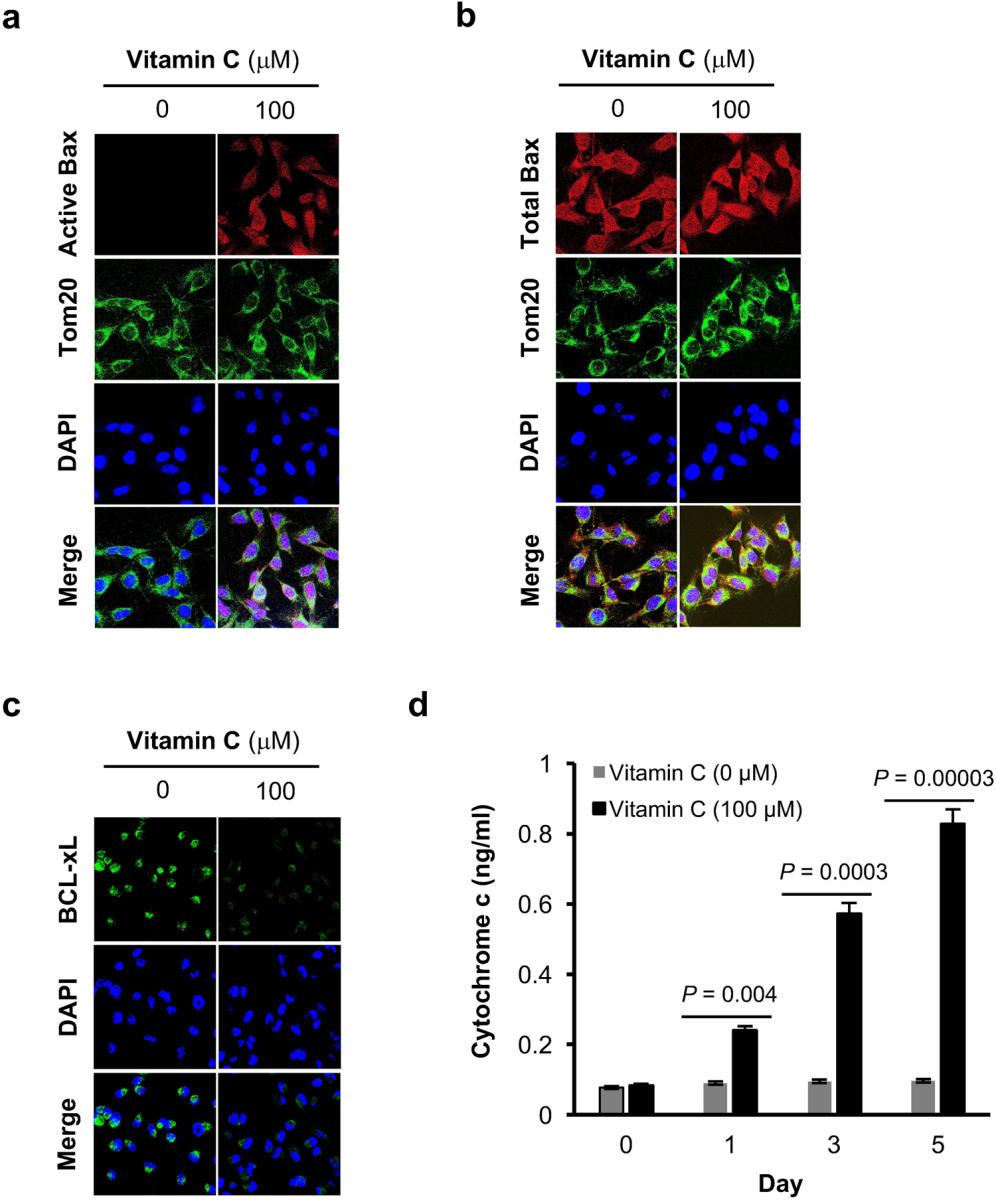
Vitamin C activates Bax, reduces BcL-xL, and increases cytochrome C release in breast cancer cells. (**a**) Immunofluorescence shows that vitamin C treatment induces active Bax protein in MDA-MB-231 cells. (**b**) Vitamin C has no effect on total Bax level in MDA-MB-231 cells shown by immunofluorescence. (**c**) Immunofluorescence shows vitamin C treatment diminishes BcL-xL in MDA-MB-231 cells. (**d**) Time course study shows cytochrome C release in response to vitamin C treatment in MDA-MB-231 cells.

Binding of TRAIL to its receptors triggers apoptotic pathway activation by recruiting Fas-associated protein with death domain (FADD), which in turn recruits Caspases and initiates the canonical apoptosis pathway. An alternative signaling pathway can also be triggered in some cells, where caspases, by activating Bax and releasing cytochrome C in mitochondria, amplify the apoptotic signal^19^. In MDA-MB-231 cells, active Bax was increased by vitamin C treatment while the total Bax remained largely unchanged (Fig. 7a,b). Furthermore, vitamin C treatment also reduced the anti-apoptotic regulator BcL-xL (Fig. 7c) and enhanced cytochrome C release in MDA-MB-231 cells (Fig. 7d). These results suggest that vitamin C induces apoptosis in breast cancer cells by increasing the expression of TRAIL, which activates Bax and caspases, reduces available Bcl-xL, and releases cytochrome c.

## Discussion

Epidemiological studies have shown an inverse association between dietary vitamin C intake and the recurrence or mortality of breast cancer. For instance, Greenlee *et al*. reported that frequent use of vitamin C supplements is associated with a decreased risk of breast cancer recurrence^20^.

Nechuta *et al*. reported that vitamin C use shortly after breast cancer diagnosis is associated with reduced mortality and recurrence risk by investigating a cohort of 4,877 women aged 20 to 75 years diagnosed with invasive breast cancer^21^. Other studies also support the benefits of vitamin C supplementation in reducing the risk, recurrence, and mortality of breast cancer^22–24^. Possibly due to the fact that dietary vitamin C is difficult to control quantitatively in human subjects (especially in control groups), the effect of vitamin C on breast cancer has not been consistent in all published studies^25^. The reasons that some studies showed “mixed or minimal benefit” of vitamin C breast cancer prevention and treatment could include the following: 1) Vitamin C supplements in treatment groups can easily be confounded by consumption of vitamin C-rich fruits and vegetables in control/placebo groups; 2) Most studies examined vitamin C consumption by questionnaire or other indirect self-report methods^25^, but did not verify effects of vitamin C levels in breast tissues or serum directly; 3) The effect of vitamin C can be complicated by other antioxidants or micronutrients. Even with these difficulties, recent meta-analyses of observational studies indicate a potential benefit of vitamin C in breast cancer survivors. One meta-analysis of epidemiological observational studies showed that higher plasma levels of vitamin C are associated with a reduced breast cancer risk^26^. Another meta-analysis (n = 17,696 breast cancer patients) showed a statistically significant association between the use of vitamin C supplements and reduced breast cancer mortality^27^. Animal experiments with the advantage to control diet tightly showed that vitamin C supplements inhibit the growth and metastasis of 4T1 murine breast cancer tumorgrafts in gulonolactone oxidase knockout (Gulo^−/−^) mice^28^, which like humans cannot synthesize vitamin C *de novo*. However, the mechanistic role of vitamin C in breast cancer treatment is not fully understood.

Different molecular mechanisms have been proposed to underlie the role of vitamin C in cancer prevention and treatment, including decreased delivery of damaging reactive oxygen species to the cell, decreased metastatic cell invasion due to enhanced collagen crosslinking in the extracellular matrix, and altered gene expression through degradation of hypoxia-inducible factors^29^. While pharmacological levels of vitamin C have been suggested as a form of cancer therapy, the EC_50_ values of vitamin C for induction of cell death were similar in immortalized non-transformed breast epithelial cells and the breast cancer cell lines tested, suggesting that very high vitamin C levels might kill both normal breast and cancer cells at least *in vitro*.

In contrast to prior studies, the present work has focused on the epigenetic regulatory role played by vitamin C in DNA demethylation, and how it may pertain to cancer treatment. Cellular 5hmC is relatively high in normal breast epithelial but decreases during malignant breast cancer progression^3–6^. The global loss of 5hmC could change genomic stability and genome-wide transcription, leading to a cascade that drives phenotypic transformation from normal breast epithelial cells to breast cancer. A means to slow, block, or even reverse the loss of 5hmC during malignant progression may ultimately open a new avenue for breast cancer prevention and treatment. Our comparison of gene expression in breast cancers and corresponding healthy breast tissue from the same patients revealed that the vitamin C transporter SVCT2 is downregulated in a majority of breast cancer cases from the TCGA dataset. Notably, TET expression was not consistently lost and indeed expression of the 3 TETs was variably increased in cancers compared to healthy breast cells. Since vitamin C is a cofactor for TETs and enhances 5hmC generation^11–15^, supplementation of vitamin C might compensate for the decreased SVCT2 expression and oppose the loss of 5hmC in breast cancer.

We show here that vitamin C at a physiological level (100 µM) dramatically increases 5hmC content in MDA-MB-231 breast cancer cells. Pharmacological levels of vitamin C are not required to increase the generation of 5hmC in the cancer cells. Thus, our *in vitro* work evaluated the epigenetic effects of vitamin C at 100 μM, a concentration readily achieved in human plasma using diet and dietary supplements.

The upregulation of global 5hmC in MDA-MB-231 cells by vitamin C treatment shifted the transcriptome of MDA-MB-231 cells and induced apoptosis. This pro-apoptotic effect of physiological vitamin C was also observed in two additional breast cancer cell lines. The induction of apoptosis by vitamin C involved and required the upregulation of TRAIL, which was one of the most significantly altered transcriptional changes observed in vitamin C-treated breast cancer cells. The increase in TRAIL expression by vitamin C correlates with the increase of 5hmC and is TETs-dependent, since knocking down TETs abolished the effect of vitamin C on TRAIL. Vitamin C treatment, mediated by TRAIL, induces apoptosis by activating Bax and caspases, decreasing Bcl-xL, and releasing cytochrome C. The effect of vitamin C at achievable physiological levels to induce breast cancer cell apoptosis by inducing TRAIL via the TET-mediated DNA demethylation warrants further preclinical and clinical evaluation as a means to prevent and treat breast cancer.

## Methods

### Cell culture and treatments

Human breast cell lines including MDA-MB-231, MCF7, ZR751, BT549, HCC1937 and human breast epithelial cell line MCF-12 were purchased from ATCC. The immortal, non-tumorigenic breast epithelial cell line (BPE3), derived from a healthy human subject, was obtained from Dr. Tan Ince (University of Miami). These cell lines were cultured in DMEM media (Sigma-Aldrich, St. Louis, MO). After seeding in 6-well plates for 24 hours, cells were treated with vitamin C (sodium ascorbate) (Sigma-Aldrich) or Glutathione (GSH, Merck Millipore, Billerica, MA) at different concentrations for varying durations. Each treatment group consisted of three wells for every experiment. Each experiment was repeated at least three times.

### Dot-blot assay

Genomic DNA was extracted from cultured MDA-MB-231 cells using QIAamp DNA mini kits (Qiagen) according to the manufacturer’s instructions. A Qubit Fluorometer (Thermo Fisher Scientific) was used to quantify the DNA. The dot-blot procedure followed methods used in our prior studies^11^. Briefly, DNA samples were diluted with 2 N NaOH and 10 mM Tris·Cl, pH 8.5, then loaded on a Hybond N + nylon membrane (Roche Diagnostics, Mannheim, Germany) using a 96-well dot-blot apparatus (Bio-Rad, Hercules, CA). After baking at 80 °C for 30 min and blocking with 5% non-fat milk for 1 hour at room temperature, the membrane was incubated in a polyclonal anti-5hmC antibody (1:10,000 dilution, Active Motif, Carlsbad, CA) at 4 °C overnight. 5hmC was visualized by chemiluminescence using ECL substrate (Thermo Fisher Scientific). The dots signal densities were captured by AlphaImager. To ensure equal loading, membranes were stained with methylene blue post-immunoblotting. Statistical significance of differences in 5hmC content between different treatments were assessed by Student *t* test, at α = 0.05.

### RNA-seq

MDA-MB-231 cells cultured in 6-well plates were treated with or without vitamin C (100 μM) for 3 days. The medium was changed daily before each treatment to avoid the accumulation of vitamin C. Total RNA was then extracted from the cells using the RNeasy Mini Kit (Qiagen). A Bioanalyzer 2000 was used to measure the quality of RNA (Agilent, Santa Clara, CA). All samples’ RNA integrity numbers (RIN) were above nine. Whole transcriptome sequencing was carried out at the Sequencing Core of John P. Hussman Institute of Human Genomics at the University of Miami using the Epicentre Ribo-Zero Human/Mouse/Rat kit (Epicentre, Madison, WI). Briefly, after ribosomal RNA (rRNA) was depleted, sequencing libraries were constructed following the standard Illumina protocols and were then processed by a Hiseq. 2000 sequencing system (125 bp paired-end reads, 4 samples per lane; Illumina, San Diego, CA). Raw read data was first run through quality control metrics using FastQC (http://www.bioinformatics.babraham.ac.uk/projects/fastqc/). Sequence reads were aligned to the human transcriptome (GRCh38, Ensembl.org) and quantified using the STAR aligner^30^. Statistical significances were determined using 2 different differential expression calculators: edgeR and DESeq. 2^31,32^. To reduce false positives, only genes with an adjusted *P*-value below 0.05 across both methods were considered differential.

### Cell proliferation assay

MDA-MB-231, MCF7, and MCF-12A cells were maintained in culture flasks at 37 °C with 5% CO_2_. The cells were seeded in white bottom 384-well plates (Thermo-scientific) at a density of 5 × 10^2^ cells per well in 25 μL medium and were allowed to attach and grow for 24 hours. Afterwards, a 5 μL solution of different concentrations of sodium vitamin C were added to each well in order to generate dose response curves from a 10-point 1:3 dilution series starting at a nominal test concentration of 0.1 μM. Each concentration was repeated in triplicate. The cells were incubated for 72 hours and then live cell counts were measured by CellTiter-Glo assay (Promega, Madison, WI) following manufacturer’s protocol. The Envision Multi-label Reader (Perkin Elmer, Waltham, MA) was used to measure the luminescence produced by the live cells. For each concentration of vitamin C, percent cell survival was plotted. The reported EC_50_ values were generated from fitted curves by solving for the X-intercept value at the 50% inhibition level of the Y-intercept value.

### Apoptosis assay

Breast cancer cells (MDA-MB-231, BT549, HCC1937) were seeded in 24 well plates with coverslips and treated with sodium ascorbate (Sigma-Aldrich St. Louis MO) at different concentrations for 5 days. Glutathione (GSH) was also used to treat cells in a control experiment at a concentration similar to the highest concentration of vitamin C. Apoptotic cells were detected at the end of the treatment utilizing the following two different techniques: (1) colorimetric TUNEL was measured by an *in situ* apoptosis detection kit (Trevigen, Gaithersburg, MD); and (2) Caspase activation was evaluated by incubating cells with FAM/FLICA Poly Caspase Detection Reagent (Biorad, Hercules, CA) per vendor’s protocol prior to imaging. Anti-TRAIL monoclonal antibody at different concentrations was added to block the effect of vitamin C on apoptosis. Caspase activity in each well was imaged using a 2D fluorescent microscope system and analyzed with ImageJ. All experiments were repeated at least 3 times.

### RNAi sequences and transfection

RNA interference (RNAi) sequences directed against human TET1 (5′-CUUUAAUGGCUGUAAGUUU-3′), human TET2 (5′-GCCUUGAGCAGUAAUAUU-3′), human TET3 (5′-AGGCCAAGCUCUACGGGAA-3′), nontarget scramble (5′-GCCUUGAGCAGUAAUAUUU -3′), were designed and synthesized by Dharmacon (Lafayettte, CO). Prior to RNAi transfection, MDA-MB-231 cells were plated in growth medium without antibiotics at 30 to 50% confluence. Transfection of RNAi sequences (10 nM concentration for each TETs, 30 nM total final concentration) was performed using Lipofectamine 2000 (Invitrogen, Carlsbad, CA), as specified by Invitrogen. Cells were maintained for 5 days (transfected on Day 0 and Day 3). Media was changed 6 hours after transfection to eliminate the toxic effects of transfecting reagents.

### Quantitative real-time RT-PCR

RNA was extracted from cultured BPE3 cells and 4 lines of breast cancer cells using RNeasy kits Qiagen, Hilden, Germany). A nanodrop 8000 spectrophotometer was used to measure the yield of RNA extraction (Thermo-Fisher Scientific, Waltham, MA). The qScript Flex (Quanta Biosciences, Beverly, MA) was used for reverse transcription (RT) according to the manufacturer’s instructions. Quantitative real-time RT-PCR (qRT-PCR) was performed in triplicate on an Applied Biosystems 7900HT using the PerfeCTa SYBR Green Fast Mix ROX (Quanta Biosciences) master mix with 10 μl reaction and 100 ng of cDNA. All primers were designed to span introns (Supplementary Table 2). The transcripts amplification results were analyzed with the Applied Biosystems software (SDS 2.4), and all values were normalized to the levels of the GAPDH using the 2^−(ΔΔCt)^ method. Statistical significance of differences in expression levels between BPE3 cells and various breast cancer cell lines, or between MDA-MB-231 cells treated with or without vitamin C were assessed by Student *t* test, at α = 0.05.

### Immunoblot

Total protein extracted from MDA-MB-231 cells was loaded onto a 4–15% gradient polyacrylamide gel (Bio-Rad) and then transferred to a PVDF membrane (Bio-Rad). After being blocked by 5% non-fat milk for 1 h at room temperature, the membrane was incubated in rabbit anti-TRAIL monoclonal antibody (1:1,000, Cell Signaling Technologies, Danvers, MA) at 4 °C overnight. TRAIL was visualized by chemiluminescence using ECL substrate (Thermo Fisher Scientific). To ensure equal loading, the membrane was stripped and reprobed by mouse anti-GAPDH monoclonal antibody (1:1,000, Santa Cruz Biotechnology, Dallas, TX) and visualized by chemiluminescence. The densities of the bands were captured by ImageJ and TRAIL levels were normalized to GAPDH. Statistical significance of differences in TRAIL content between different treatments were assessed by Student *t* test, at α = 0.05.

### ELISA

Conditioned cell culture media was removed after three days of incubation with MDA-MB-231 cells. Vitamin C was added at 100 μM only at the initial treatment. Total levels of TRAIL protein in cell culture media were measured using ELISA test kits (R&D Systems, Minneapolis, MN) according to the manufacturer’s instructions. Briefly, 150 μl of cell supernatant was added to each well and incubated overnight at 4 °C with gentle shaking. Wells were aspirated and washed and then incubated with TRAIL conjugate for 2 hours at room temperature. Wells were again washed and incubated with substrate for 30 minutes at room temperature. Following addition of stop solution to curtail development of substrate, plates were read at 450 nm with wavelength correction at 570 nm. Statistical significance of differences in TRAIL content between different treatments was assessed by Student *t* test, at α = 0.05.

### Quantification of Cytochrome C

MDA-MB-231 cells were treated with vitamin C (100 μM) for 1, 3 or 5 days. Equal number of cells (1.5 × 10^6^) were collected from each treatment point and Cytochrome c was measured using Cytochrome c Human ELISA kit (abcam, Cambridge, UK). For control (0 days), cells were collected immediately after adding vitamin C.

### Immunofluorescence

MDA-MB-231 cells were seeded in 6-well culture dishes with coverslips for 24 hours before treatment. After completion of treatment, coverslips with cells were washed three times with cold PBS. The cells were fixed for 10 minutes at room temperature with 4% paraformaldehyde in PBS, permeabilized for 5 minutes with 0.2% Triton X-100 PBS, and blocked by 5% BSA. The cells were then incubated with the primary antibodies (Active Bax: 6A7 (Santa Cruz, Dallas, TX), Total Bax: Bax (2D2) (Santa Cruz, Dallas, TX), and Bcl-xL: 2762 (Cell signaling, Danvers, MA) at 1:50 dilution in PBS over night at 4 °C, followed by the secondary antibodies at 1:250 dilution in PBS for another hour. Each step was preceded by three washes in PBS. To stain the nucleus, cells were incubated with 40 µg/ml 4′,6-diamidino-2-phenylindole (DAPI) for 20 minutes at room temperature. The coverslips were then mounted on glass slides and examined at room temperature with a Zeiss LSM 710 confocal laser scanning microscope. Images were processed with the help of Merge-color application of NIH ImageJ software.

### Statistical analysis

All data were normalized to inner controls, such as GAPDH expression level. Data were presented as mean ± standard error of the mean (S.E.M.). Statistically significant changes amongst treatments were assessed by Student *t* tests at α = 0.05.

## Electronic supplementary material

Supplementary Information
