# Supplementary material for: Vitamin C promotes apoptosis in breast cancer cells by increasing TRAIL expression
Source: Sci Rep. 2018 Mar 28;8:5306. doi: 10.1038/s41598-018-23714-7 (PMC5871772; doi:10.1038/s41598-018-23714-7)
Supplement: Supplementary file 1 — Supplementary Information [file 41598_2018_23714_MOESM1_ESM.docx]

**Supplementary Information**

**Vitamin C promotes apoptosis in breast cancer cells by increasing TRAIL expression**

David W. Sant^1*^, Sushmita Mustafi^1*^, Christopher B. Gustafson^1^, Joshua Chen^1^, Joyce M. Slingerland^2,3^, Gaofeng Wang^1, 3§^

^1^ John P. Hussman Institute for Human Genomics, Dr. John T. Macdonald Foundation Department of Human Genetics; ^2^ Braman Family Breast Cancer Institute; ^3^ Sylvester Comprehensive Cancer Center; University of Miami Miller School of Medicine, Miami, FL 33136, USA.

^§^ Correspondence should be addressed to: Gaofeng Wang, Ph.D.,

**Supplemental Table 1. Differentially expressed genes in MDA-MB-231 cells caused by vitamin C treatment**

| **Symbol** | **Gene** | **Major Function** | **RNA-seq (edgeR)** | |  | **qRT-PCR** | |
| --- | --- | --- | --- | --- | --- | --- | --- |
|  |  |  | **Fold** | ***P* value** |  | **Fold** | ***P* value** |
| TNFSF10 | Tumor Necrosis Factor (Ligand) Superfamily, Member 10 | apoptosis | 1.87 | 9.3 x 10^-7^ |  | 2.18 | 0.0018 |
| CYP1B1 | Cytochrome P450, Family 1, Subfamily B, Polypeptide 1 | drug metabolism | 1.93 | 3.3 x 10^-19^ |  | 1.96 | 0.0361 |
| TFRC | Transferrin Receptor | iron transportation | 0.69 | 0.0001 |  | 0.53 | 0.0242 |
| PGK1 | Phosphoglycerate Kinase 1 | angiogenesis | 0.64 | 1.3 x 10^-8^ |  | 0.60 | 0.0075 |
| BNIP3 | BCL2/Adenovirus E1B 19kDa Interacting Protein 3 | apoptosis | 0.44 | 1.4 x 10^-26^ |  | 0.59 | 0.0342 |
| NDRG1 | N-Myc Downstream Regulated 1 | cell growth and differentiation | 0.39 | 4.0 x 10^-13^ |  | 0.37 | 0.0006 |
| BNIP3L | BCL2/Adenovirus E1B 19kDa Interacting Protein 3-Like | apoptosis | 0.63 | 6.4 x 10^-6^ |  | 0.62 | 0.0303 |
| ADM | Adrenomedullin | angiogenesis | 0.42 | 1.2 x 10^-19^ |  | 0.54 | 0.0052 |
| PDK1 | Pyruvate Dehydrogenase Kinase, Isozyme 1 | homeostasis of carbohydrates | 0.50 | 1.1 x 10^-16^ |  | 0.49 | 0.0094 |
| HK2 | Hexokinase 2 | glucose metabolism | 0.51 | 5.5 x 10^-7^ |  | 0.46 | 0.0051 |

**Supplemental Table 2. Primers for quantitative real-time RT-PCR**

| **Gene** | **Forward (5′→3′)** | **Reverse (5′→3′)** |
| --- | --- | --- |
| TET1 | AATGGAAGCACTGTGGTTTG | ACATGGAGCTGCTCATCTTG |
| TET2 | AATGGCAGCACATTGGTATG | AGCTTCCACACTCCCAAACT |
| TET3 | GAGGAGCGGTATGGAGAGAA | AGTAGCTTCTCCTCCAGCGT |
| SVCT1 | TCATCCTCTTCTCCCAGTACCT | AGAGCAGCCACACGGTCAT |
| SVCT2 | TCTTTGTGCTTGGATTTTCGAT | ACGTTCAACACTTGATCGATTC |
| TNFSF10 | GCTGAAGCAGATGCAGGACAAG | GCTGACGGAGTTGCCACTTGAC |
| CYP1B1 | CTCACCGACCCCCAGTCTCA | GTTTAGCGGCCAAGGGTCGT |
| TFRC | CGGAGGACGCGCTAGTGTTC | GCCGGTGAAGTCTGTGCTGT |
| PGK1 | CTTGGAGAGCCCAGAGCGAC | CACAGTCCAAGCCCATCCAGC |
| BNIP3 | TCTCACTGTGACAGCCCACCT | CGGCCGACTTGACCAATCCC |
| NDRG1 | CTCCCAGCCGGCCAAGCT | GGTGCCATCCAGAGAAGTGACGCTG |
| BNIP3L | GTTGTGTTGCTGCCTGAGTGC | AGCTCCACCCAGGAACTGTTG |
| ADM | ACGGAAACCAGCTTCATCC | GCCAGTGGGACGTCTGAG |
| PDK1 | GGCAAAGGAAGTCCATCTCATCG | GCTGTCCTGGTGATTTTGCATTTAGTTC |
| HK2 | GACCAAGTGCAGAAGGTTGACCAG | GTACCTGTCCCATCTGGAGTGG |
| GAPDH | TGGACCTGACCTGCCGTCTA | CCCTGTTGCTGTAGCCAAATTC |

**Supplementary Figure 1**


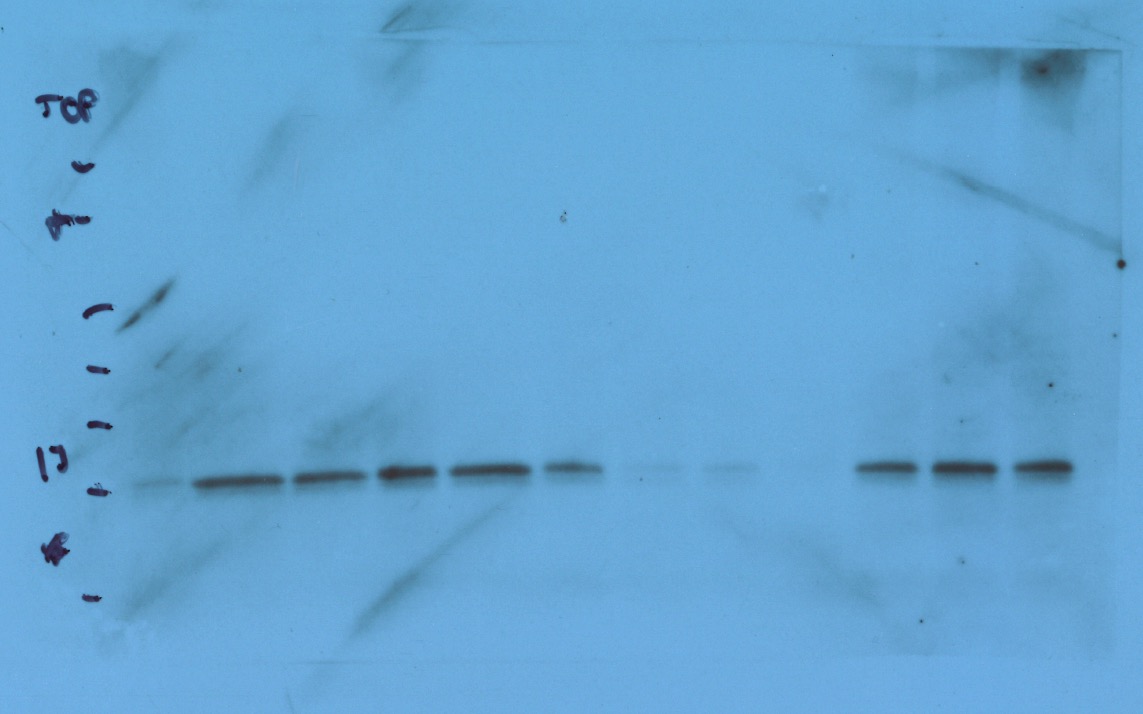

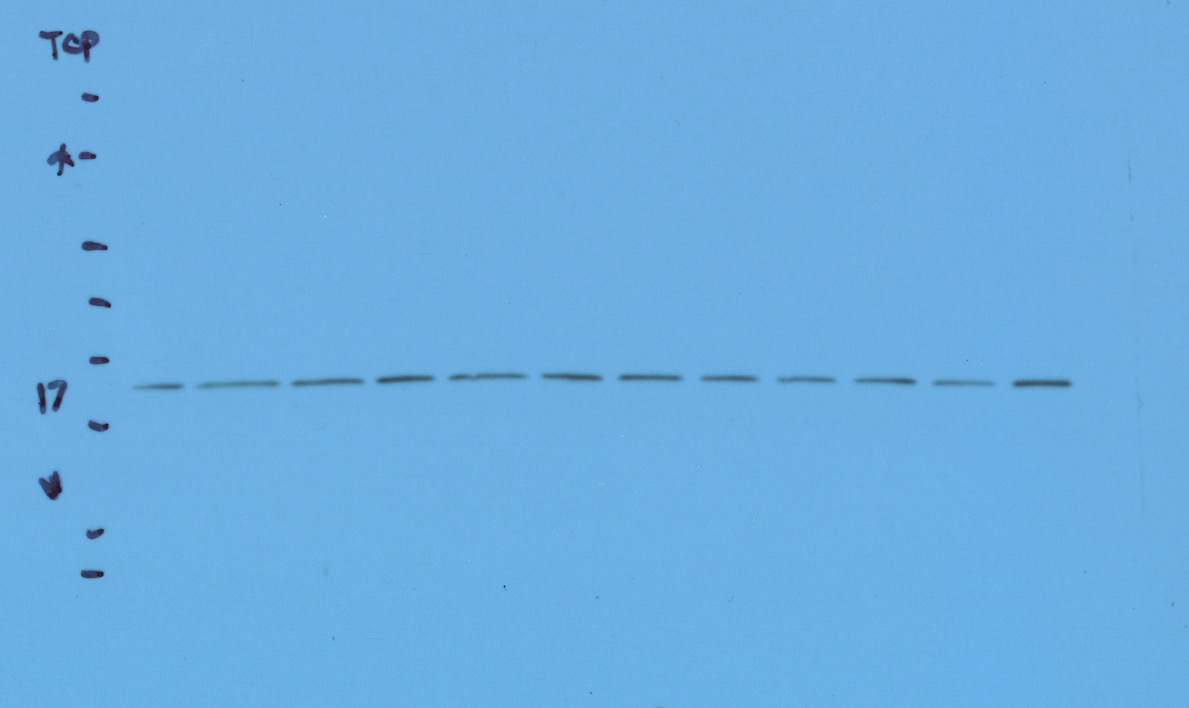


0

Vitamin C (μM)

TRAIL

GAPDH


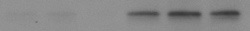

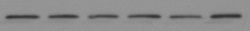


100

0

Vitamin C (μM)

100

0

Vitamin C (μM)

100

GAPDH (~36kDa)

TRAIL (~30kDa)

**A**

**B**

**Supplementary Figure 1.** Immunoblot membrane images of TRAIL expression in MD-MBA-231 cells treated with (100 μM) or without vitamin C. The cropped images are shown in **Figure 4b**. The others lanes are irrelevant to vitamin C experiment presented here. **(A)** Immunoblot membrane was probed with anti-TRAIL primary antibody and was imaged on X-ray film. Note that lanes 1-6 of the membrane were for an unrelated experiment. **(B)** Immunoblot membrane was re-probed with anti-GAPDH primary antibody and was imaged on X-ray film. Note that lanes 1-6 of the membrane were for an unrelated experiment.

**Supplementary Figure 2**

**
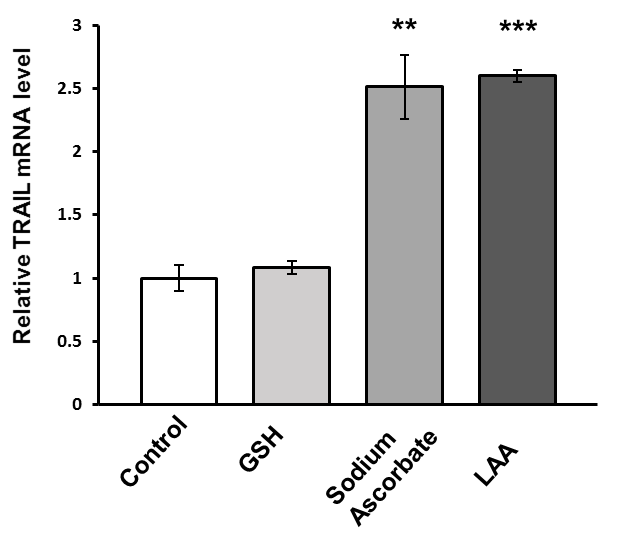
**

**Supplementary Figure 2. Vitamin C, not glutathione, increases the expression of TRAIL.** The mRNA levels of TRAIL measured by qRT-PCR are increased in MDA-MB-231 cells treated for 5 days with 100 µM sodium ascorbate or 100 µM L-ascorbic acid, but not with 100 µM glutathione (GSH) compared to non-treated controls. (** *P* < 0.01, *** *P* < 0.001).

**Supplementary Figure 3**

**
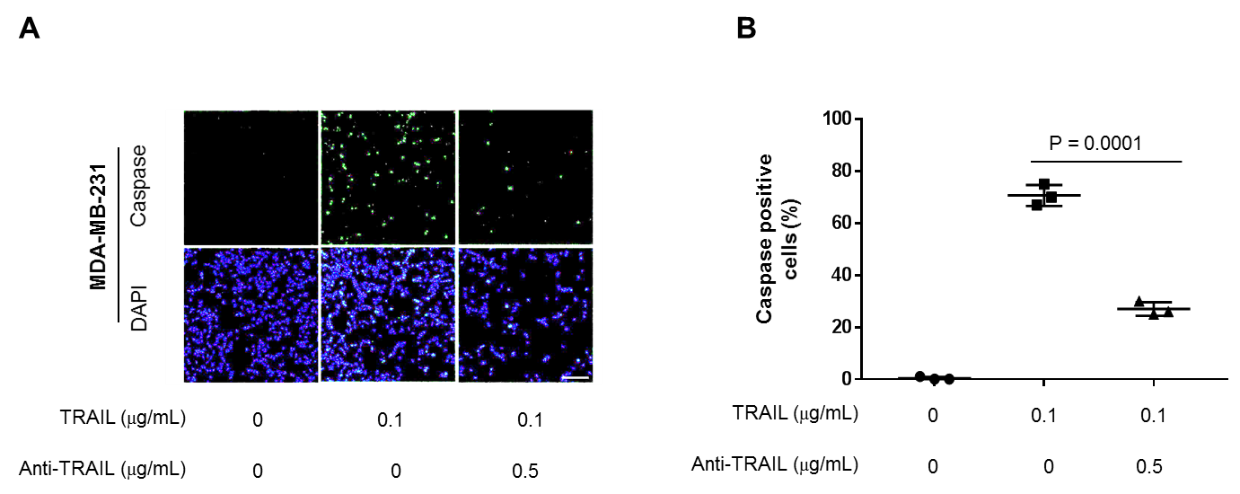
**

**Supplementary Figure 3. TRAIL protein induces apoptosis.** Caspase staining **(A)** and quantification **(B)** show that addition of TRAIL protein (0.1 μg/mL) to the cell culture media induced apoptosis in ~75% of MDA-MB-231 cells, but this effect was largely abolished by the addition of anti-TRAIL antibody to the media (*P* = 0.0001).
